# Attitudes of Pig and Poultry Industry Stakeholders in Guandong Province, China, to Animal Welfare and Farming Systems

**DOI:** 10.3390/ani9110860

**Published:** 2019-10-24

**Authors:** Michelle Sinclair, Wang Yan, Clive J. C. Phillips

**Affiliations:** 1 Centre for Animal Welfare and Ethics, School of Veterinary Sciences, The University of Queensland, Gatton, QLD 4343, Australia; c.phillips@uq.edu.au; 2 College of Animal Science, South China Agriculture University, Guangzhou 510642, China; ywang@scau.edu.cn

**Keywords:** China, pig, poultry, stakeholder, survey

## Abstract

**Simple Summary:**

The People’s Republic of China produces large numbers of animals for food but little is known about the attitudes of people employed in the industries towards the welfare of animals in the farming systems. We surveyed attitudes of people involved in the pig and poultry industries in the Guandong Province of China towards the welfare of their animals. They generally supported improving welfare and believed outdoor systems to be better for welfare and intensive indoor systems to be better for food safety. Farmers using intensive, indoor farming systems showed less enthusiasm to improve welfare than those using outdoor systems. Chicken farms without enrichment were perceived to be particularly in need of welfare improvement, and this was most likely to happen on small farms.

**Abstract:**

Although the People’s Republic of China produces more animals for consumption than any other country, very little is known about the attitudes of stakeholders in the livestock industries to animal welfare in farming systems. This study investigated the attitudes of stakeholders in pig and poultry farming in south China towards animal welfare in different farming systems, pig and poultry behaviour, and the inherent value of the animals themselves. Respondents thought welfare was important, particularly if they had worked in the industry a long time, and that they intended to make improvements, even though they also believed it to be generally satisfactory. Outdoor systems were perceived to be better for welfare but indoor systems better for food safety, particularly among respondents that had gained their knowledge from multiple sources. Respondents believed pigs and chickens to have equally important needs, despite the fact that pigs were considered more intelligent than chickens. Pig farmers with outdoor systems had a more positive attitude to making welfare improvements compared with those operating intensive indoor systems. However an absence of enrichment in chicken farms increased respondents’ intentions to make improvements, and these were more likely to occur on small chicken farms. Veterinarians and government officials were more likely to perceive welfare as unsatisfactory or to want change it than those working directly with animals. City residents were more likely to support and express confidence that they could improve animal welfare, compared to rural residents. It is concluded that stakeholders in China’s pig and poultry production industries recognised a need to improve welfare, although they saw a conflict with production of safe food. However, farmers involved in intensive production systems were less likely to perceive a need or capacity to improve welfare than those operating more extensive systems, suggesting a dichotomisation of the people in the industry into those in small and outdoor farms that could and were improving welfare and those in indoor intensive farms who did not envisage this happening.

## 1. Introduction

The People’s Republic of China (henceforth China) is the largest producer of meat from terrestrial livestock in the world, with approximately 14 billion animals raised and slaughtered in 2017. The scale of livestock agricultural output in China exceeds that of the world’s second largest producing country (United States of America) by over 36%, and over 57% more than the third (Brazil), with the United Kingdom, France and Germany producing less than 10% of China’s output [1]. The scale and global importance of China’s livestock industry is not limited to terrestrial animals, with the nation currently producing a majority of the world’s farmed fish (63 million tonnes), approximately four times the output of the second largest producer, Indonesia [2]. With over 1.4 billion citizens [3] and continued domestic growth, China also supports the world’s largest food consumption by volume [4]. Considering these details, China’s potential influence on global animal welfare is undeniable.

**Figure 1:**
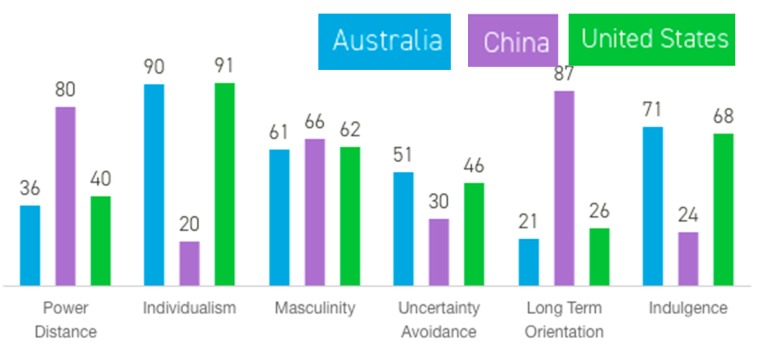
Comparison of cultural dimensions between China and ‘western’ countries, Australia and the United States. Data and graph from ‘Hofstedes Insights’ (www.hofstede-insights.com [5]).

Despite the size and status of China, and its dominance in global agriculture, Chinese animal welfare laws do not yet exist, and very little is known about the Chinese livestock industry outside of China, and even less about Chinese farmers. Subject to a farming environment that differs both within the country and with that in other countries, cultural differences provide another impediment to a deeper understanding of Chinese livestock communities. The difference between measurable cultural dimensions is particularly large when comparing China with countries with which it has major economic relationships, such as the European Union, Australia and the United States. These differences are primarily on the dimensions of ‘power distance’ (the extent to which less powerful members of society expect and accept inequality in power distribution) in which China ranks high while US, UK and Australia rank low; ‘individualism’ (the extent to which community members take care of themselves independently [the ‘I’] rather than for a wider collectivist group [the ‘we’]), in which China ranks low, with US, UK and Australia ranking high; ‘long term orientation’, in which China ranks high, with US, UK and Australia ranking low, and lastly, ‘indulgence’, the extent to which people indulge their impulses rather than show restraint, on which China ranks low, with US, UK and Australia ranking high (see Figure 1) [5]. This has important implications for international agricultural trade relationships, and for proponents of international animal welfare initiatives. Given the possibility of miscommunication and misunderstanding resulting from these key cultural differences, the need to better understand agricultural trading partners is paramount, particularly concerning the growing issue of animal welfare.

Previous research shows a need to engage livestock stakeholders in international animal welfare initiatives, through the identification of mutual benefits, and emphasises the role of local knowledge, understanding and respect in success [6]. To do this, the farmers themselves need to be consulted, and their position, environment, and attitudes adequately understood.

A study with French pig farmers found that their understanding of animal welfare correlated with their levels of involvement in consumer quality assurance programs [7]; farmers participating in stringent quality assurance schemes defined animal welfare in terms of providing environments in which the animals can perform their natural behaviours, with farmers in less stringent systems defining animal welfare as simply the physiological health of the animals. A correlation of farmers’ attitudes with the level of regulation and comprehensiveness of animal welfare requirement was also evident in a literature review incorporating several studies in neighbouring European countries [8]. Another study found that Dutch pig farmers’ general attitudes towards animal welfare was tied to their understanding of good farming practices, which were primarily influenced by the market segment they were targeting [9]. Likewise, in a study of German pig farmers, attitudes towards animal welfare, and their willingness to participate in animal welfare programs, was most prominently tied to economic factors and the ability to increase profit, and a framework for an incentive scheme was suggested [10]. Another European study found a relative consensus between the expectations of industry stakeholders and consumers on the elements that are required to satisfy animal welfare from 72 attributes of the farming process [11]. However, it was only achieved if welfare was included into the broader concept of quality, and managed by assurance and sustainability schemes [11].

Most investigation of farmers’ attitudes has so far been conducted in Europeans countries, with very little research of this nature conducted in an Asian context. In China, although little research has been conducted with farmers and other livestock stakeholders, studies have investigated citizens’ attitudes to animal welfare. One found that only two thirds of respondents had heard of ‘animal welfare’, but when they knew what it was 73% were in favour of improving it for human food safety reasons, with 66% agreeing to some extent that animal welfare legislation should exist in China, where it currently doesn’t [12]. This work shows that animal welfare may be a concept of influence in China, at least amongst Chinese citizens. Likewise, when ‘animal protection’ was measured against twelve other world social issues, such as poverty, gender inequality and racism, it was rated by university students in China as the most important issue, alongside environmental protection and sustainable development [13]. Additional studies have begun to investigate attitudes in focus groups of Chinese industry stakeholders, in which the importance of economic factors, mutual benefits and financial incentives connected to welfare, such as improved animal productivity, food safety, and above all, quality, were demonstrated [14]. Chinese stakeholders have indicated that the law, governance, the availability of tools and resources, and knowledge are important drivers behind their attitudes to animal welfare, and the workplace hierarchy and the extent to which animal welfare improvements were approved of within the workplace was perceived as highly influential in regarding ability to improve welfare in China [15]. This confirms the importance of the ‘power distance’ cultural dimension in Chinese society. A quantitative survey has investigated stakeholders’ perceptions of important animal welfare concerns during transport and slaughter [16], with the absence of stunning pre-slaughter being considered the most serious threat to animal welfare.

This study aims to build on an earlier survey study that investigated attitudes to animal welfare of stakeholders involved in slaughter and transport [15], by focussing on stakeholders in the farming systems, including investigation of their attitudes to the animals. Poultry and pork producing farms were the focus, given the significance of these species in China, which is home to half of the global population of pigs [17], and the largest chicken production industry in the world, double that of their closest competitors (Indonesia and the United States) [18].

## 2. Materials and Methods

Under the auspices of the Animal Welfare Standards Project (AWSP; www.animalwelfarestandards.org), 16 workshops on improving animal welfare on farms were held in southern China from June to September 2018, within which 178 farmer participants completed a survey. Participants were required to attend the workshops by their employer, which were hosted by college facilitators. Although the survey was not compulsory for workshop participants, 100% of participants that were issued surveys chose to complete them. Although an estimate of the total people engaged in pig and poultry farming in this region is difficult to find, The Guangzhou Municipal Government estimated 788,000 people work in agricultural, forestry, husbandry and fisheries industries. Estimating that one quarter of these workers are engaged in pig and poultry agriculture (197,000) in the region, the sample represents 0.9% of total workers [19]. The locations of the workshops were all within Guangdong province in south-east China, including Guangzhou (n = 32 farmers), Huizhou (n = 34), Jiangmen (n = 34), Maoming (n = 21), Shenzhen (n = 34), and Yunfu (n = 23). This province hosts some of the largest pig farming enterprises in the world [20].

Workshops were organised by 16 facilitators, who were senior livestock leaders within the localities of the workshops. Facilitators were selected by academic staff in the South China Agricultural University (SCAU), Guangzhou, Guandong Province, and had to have enough knowledge and experience to train effectively and to be sufficiently senior within the pig or poultry farming industry to encourage attendance at the workshops they hosted. They had to have previously attended a four-day training workshop series at SCAU in March of the same year, delivered by international and Chinese animal welfare experts. Facilitators were provided with the present survey and administered it before commencement of each workshop. Because of potential difficulties in translating the term ‘animal welfare’ into Chinese, at the start of the survey respondents were provided with a definition of animal welfare, modified from that provided by the OIE: ‘The term *animal welfare* refers to how well an animal is coping with the conditions in which it lives. An animal has good welfare if its needs are being met and hence it is healthy, comfortable, well nourished, safe, able to express important behaviour and not suffering from unpleasant states such as pain, fear and distress’ [21]. The survey was based on a previously tested survey tool that aimed to investigate attitudes to animal welfare, along with motivations and barriers to improving animal welfare, and had been used by the same researchers in previous studies [15,22,23]. Some targeted attitude questions were added that sought to identify farmers’ attitudes to various welfare-related components of pig and poultry farming systems, whichever was relevant, and to the animals themselves (see survey items in appendix). The survey was translated into Mandarin and back translated by the team at SCAU to ensure accuracy.

Facilitators were provided with a USB drive containing the translated survey for printing and paper-based distribution within the workshops, along with translated training resources for use within the workshops to follow. Workshop participants were invited by the facilitators based on a set of selection criteria that included current employment within the poultry and pig farming industry and being aged over 18. The survey instrument was reviewed by social scientists, as well as having been mostly validated in earlier studies [15,22], with the novel animal and farming systems based questions refined to ensure comprehension, relevance and accuracy. Ethical approval for this study was granted by the University of Queensland Human Ethics Committee (Project Identification Code: 2017000628).

### Statistical Analysis

The data were initially collated and quality controls used to remove obviously erroneous data points. Data was imported into Minitab and initial descriptive statistics were obtained, with means, for each of the general attitude questions, in addition to the attitudes to farming systems and the attitudes to the pig and chickens. Attitude responses were used in a general linear model (GLM), and the numbers 1–5 were attributed to the scaled responses ‘strongly disagree’ to ‘strongly agree’ to facilitate this process.

Ordinal logistic regression analyses were used to assess the significance of the relationships between respondent demographics (the categorical independent variables) and the distribution of the Likert scale responses for each of the attitude questions (the continuous dependent variable). The model used an iterative-reweighted least squares algorithm with a logit link function. All models achieved convergence and results are presented as Odds Ratios (OR) and Confidence Intervals (CI). All probability values were considered significant at *p* < 0.05, and highly significant at <0.01.

## 3. Results

### 3.1. Demographics

Male stakeholders represented 59% of the respondents, females 41% (Table 1), which indicates over-representation of males compared with national data (approximately 53% male, 47% female [23]). Most lived in urban (60%) or rural (40%) zones, the former being similar to the national distribution of urban-zoned residents (59.2% [23]). The majority (71%) were aged 26–45, whereas this age group represents only 50% of the population of China, which overall is older [24]. The most common work was directly with animals, although a significant proportion were business owners or managers. 

The farming systems represented were mainly intensive indoor pig farming, most commonly with over 3000 pigs, and about one third were also involved in indoor chicken production, many keeping just small numbers of birds, but also some with large flocks of over 10,000 birds. Most said that they had gained their knowledge from employment in agriculture or through formal qualifications, and to a lesser extent personal interest. Nearly half had been involved in the industry for less than one year, with only a small proportion (20%) having ten years or more experience in the industry.

### 3.2. Attitudes to Pigs and Chickens in Farming Systems

There were differences in the level of agreement with the different questions about respondents’ attitudes to animals (Table 2). Respondents were most in agreement that welfare of animals was important to them and that they intended to make improvements to animal welfare. There was slightly less agreement that they had tried in the past or that they could make improvements, and still less that important others would approve of them making improvements. There was least agreement that welfare was satisfactory in their workplace.

Concerning respondents’ attitudes to different types of pig and chicken production systems and their impact on welfare, respondents mostly agreed that outdoor pig and chicken production systems, where the animals were in groups and able to roam, were better for the welfare of the animals (Table 3). There was no differentiation between the two species. There was less agreement that indoor intensive systems were better for food safety, and that individual stall housing was better for sows and that it is desirable to house pigs in groups. Respondents neither agreed nor disagreed that indoor intensive systems are better for the welfare of pigs and chickens.

When considering respondents’ attitudes towards pigs and their behaviour (Table 4), respondents most readily agreed that the physical health of pigs is important, that it is also important that they are happy, and that they are intelligent animals. There was also general agreement, but less so, that they are social animals, feel pain, can suffer and should be allowed to make a nest before parturition. There was some but not overwhelming agreement that they should be allowed to forage for food and root in the ground, and even less that they are aggressive animals. Respondents tended towards disagreement that it is normal for pigs to display mouthing behaviours such as biting bars and chewing (without food) repetitively, and generally disagreed that they do not understand much about their environment, that they don’t mind if their tails are docked and teeth clipped and that they are unfriendly animals and do not like to interact with other pigs.

When considering respondents’ attitudes towards chickens and their behaviour (Table 5), respondents most readily agreed that they should be physically healthy and happy and allowed to perch, stretch and preen, as well as agreement that they can feel pain. There was some but not overwhelming agreement that they were social animals and that they should be allowed to flap their wings and make a nest, bathe in dust or dirt, and that they are intelligent animals or that they could suffer. Respondents tended to disagree that it was normal for them to attack each other or peck each other’s feathers out, and there was some agreement that chickens did care about their environment and that they are friendly and like to interact with other chickens.

Comparing responses for pigs and chickens, there were no differences between mean responses in relation to whether intensive farming is better for food safety (3.28, 3.30, *P* = 0.87), indoor intensive systems are better for welfare (3.05, 3, *P* = 0.56), outdoor systems are better for welfare (3.82, 3.80, *P* = 0.79), they can suffer (3.66, 3.53, *P* = 0.66), feel pain (3.78, 3.88, *P* = 0.16), should be happy (3.90, 3, *P* = 0.66), physically healthy (4.01, 4.02, *P* = 0.87), it is normal for mouthing/chewing repetitively (pigs) or pecking each others’ feathers out (chickens) to occur (2.94 and 2.89, respectively, *P* = 0.66) and whether pigs or chickens are more social (3.71 and 3.69, *P* = 0.72). However, respondents were more likely to agree that pigs (3.89) are intelligent than to agree that chickens are intelligent (3.68, *P* = 0.007).

### 3.3. Demographic Influences

#### 3.3.1. Role in Industry

A greater proportion of the government officials that responded (38%) thought the welfare of animals was very important to them than the proportion of those working directly with animals that responded (11%) (OR 0.06, CI 0.01–0.34, *P* = 0.001). Similarly, fewer of the veterinarians (20%) were likely to agree that the welfare of farm animals was satisfactory in their workplace, compared to those working directly with animals (40%, OR 5.86, CI 1.1–31.8, *P* = 0.04), but fewer veterinarians were also likely to agree (10%) that important others would approve of them making welfare improvements, compared to those working directly with animals (57%, OR 6.47, CI 1.1–38.3, *P* = 0.04).

#### 3.3.2. Type of Farming System

More pig farmers whose pigs were kept in either mixed indoor/outdoor systems (25%) or outdoor in paddocks (25%) agreed that important others would approve of them making welfare improvements, compared to those working directly with animals (6%) (OR 0.20 and 0.21, CI 0.06-0l.63 and 0.07–0.63, *P* = 0.006 and 0.006, respectively). More pig farmers whose animals were outdoor in paddocks (25%) were also likely to agree that they intended to make welfare improvements than those whose pigs were in intensive housing systems (9%) (OR 0.28, CI 0.09–0.88, *P* = 0.03). More pig producers operating indoor/outdoor systems (30%) and those with fully outdoor systems with paddocks (29%) strongly agreed that they were confident that they could make welfare improvements than those operating intensive indoor systems (6%) (OR 0.24 and 0.18, CI 0.07–0.81 and 0.05–0.57, *P* = 0.02 and 0.004, respectively). More pig producers operating fully outdoor systems with paddocks (21%) strongly agreed that they were confident that they had in the past made welfare improvements than those operating intensive indoor systems (6%) (OR 0.19, CI 0.06–0.62, *P* = 0.006).

More chicken producers operating intensive systems without enrichment (86%) agreed or strongly agreed that they intended to make welfare improvements than those with enrichment (53%) (OR 0.25, CI 0.07–0.84, *P* = 0.02). Fewer (46%) of the very large farmers (>10,000 birds) agreed or strongly agreed that they intended to make welfare improvements than farmers with <1000 chickens (88%) (OR 1.53, CI 1.09–2.15, *P* = 0.01).

#### 3.3.3. Time in Industry

The longer the time stakeholders had spent in industry, the more likely they were to say that the welfare of the animals is important (OR 2.27, CI 1.23–4.19, *P* = 0.009), but less likely to have tried to make changes (OR 0.41, CI 0.18–0.91, *P* = 0.03), and also less likely to agree that it is normal for pigs to display stereotypical mouthing behaviours (OR 0.37, CI 0.22–0.63, *P* < 0.001).

#### 3.3.4. Residential Zone

Residential zone was the most frequently significant demographic influence in this study, with stakeholders living in city centres more likely to strongly agree that animal welfare is important to them (41.5% of city residents), as compared to those living rurally (11.3% of rural residents) (OR 0.27, CI 0.13–0.58, *P* = 0.001). City residents were also more likely to strongly agree that they were confident they could improve animal welfare (21.5% of city residents), compared with rural residents who were far more likely to ‘agree’ (52.1% of rural residents) than ‘strongly agree’ (7%) (OR 0.18, CI 0.07–0.49, *P* = 0.001). Rural residents were more likely to ‘agree’ (49.3% of rural residents) that it is desirable to house pigs in a group situation, compared to city residents who were most likely to ‘neither agree or disagree’ (OR 02.50, CI 1.42–4.40, *P* = 0.002). City residents were more likely to ‘strongly agree’ (27.7% of city residents) that it is important that chickens are happy, compared to rural residents (15.5% of rural residents), most of whom were only likely to ‘agree’ (69%), as did suburban residents (84.2%) (OR 5.05, CI 1.52–16.8, *P* = 0.008). City residents were more likely to ‘strongly agree’ (20.0% city residents) than rural residents (7%) that chickens should be allowed to make a nest to lay eggs, however most of the stakeholders in both residential zones ‘agreed’ in general (OR 0.22, CI 0.07–0.68, *P* = 0.009). Likewise, when asked if chickens should be allowed to flap their wings, city residents again ‘strongly agreed’ (24.6%) as compared to rural residents (5.63%), again stakeholders from both residential zones were most likely to ‘agree’ (56.9% and 61.9%, respectively) (OR 0.12, CI 0.03–0.42, *P* = 0.001).

#### 3.3.5. Route of Industry Knowledge

How stakeholders gained their existing knowledge also had highly significant relationships with attitudes to pigs and chickens. Stakeholders who had gained most of their knowledge from all the suggested routes (qualifications, experience, personal interest, friends and acquaintances) were more than twice as likely to ’agree’ that intensive indoor raising of pigs is better for food safety (47.4%), compared to those who only had either a formal qualification (20%), and those who only learnt through experience on farm (19.4%). Those with a mixture of formal qualifications and experience were almost as likely to ‘agree’ as those who gained their experience by all routes (42.1%) (OR 0.35, CI 0.22–0.55, *P* < 0.001). The same question for chickens showed a similar trend, with those respondents with knowledge gained from all suggested routes being more likely to ‘agree’ that intensive indoor raising of chickens was better for food safety (39.5%) as compared to those who learnt through a formal qualification (20%); however, those who learnt on farm were even more likely to agree with this statement (45.2%) (OR 0.48, CI 0.32–0.71, *P* < 0.001).

## 4. Discussion

### 4.1. Demographics

The over-representation of males in this survey compared with national data may reflect a gender difference in senior roles within the livestock industry. Nevertheless, there are more women (19% of all women in China) than men (14% of all men in China) working in agriculture [25]. The younger age of our sample compared with national data may reflect an attraction of younger stakeholders to our training and educational workshops.

### 4.2. Perception of Pigs and Poultry in Farming Systems

Stakeholders involved in pig and poultry farming generally agreed that the welfare of the animals is important to them. In a recent survey in our selected region stakeholders in livestock transport and slaughter perceived that others would approve of them making improvements to welfare more than our stakeholders did (3.88 vs. 3.64) [15]. This could be reflective of a greater recognition of welfare issues in transport and slaughter than at the farm. Beliefs that animal welfare is satisfactory in the workplace in which they operate and that they intended to make improvements were both slightly higher for stakeholders in this study than in the previous study (means welfare satisfactory 3.36 vs. 3.18, intentions 3.79 vs. 3.58). A failure to intend to make changes may be because during slaughter a disconnection, or ‘de-animalisation’ and ‘compartmentalisation’ towards the animals as individuals with a welfare status is required in order to maintain the mental wellness of the stakeholders [26]. It could also be reflective of the limited time which slaughter stakeholders spend with individual animals, as opposed to those involved in farming, who are by nature of their goals invested in the physical wellbeing of their animals over a longer period of time.

Stakeholders most strongly agreed that ‘it is important that pigs are physically healthy’, and likewise for chickens. This probably reflects the loss of animal productivity that can occur if the animals are not physically healthy. It may also relate to a focus on food safety, on which there is a strategic focus by the Chinese government and the World Health Organisation [27]. A recent survey of Chinese nationals found that ‘food safety’ was the number one concern they would like the government to attend to [28], and focus group studies with Chinese stakeholders have found that improved food safety, along with product quality, are the most important perceived benefits of addressing animal welfare [14]. Moreover, consumers in European countries, United Kingdom, Italy and Sweden, have been shown to associate animal welfare with the healthfulness of animal products, indicating a potential parallel in perception [29]. The concern for the ‘happiness’ of the animals was only slightly less than concern for their physical health, and they exhibited preference for natural behaviour expression, both suggesting that the fundamental and practical notions that underlie animal welfare are well grasped by Chinese stakeholders, even if the ‘concept’ or term ‘animal welfare’ as it is known in western countries may not be [6].

While intensive systems were advocated for food safety reasons in our study, some of the highest levels of agreement appeared in response to the ‘happiness’ of the animals, and that outdoor roaming farming systems that allow social interaction were better for the welfare of the animals. It was believed that some natural behaviours, such as stretching, preening, perching and wing flapping should be allowed in the farming of chickens, and likewise, that pigs are intelligent animals who should be allowed to make a nest, and forage for food. This may be reflective of the preference for natural and fresh produce, in a country where knowledge of product characteristics is important to discerning Chinese consumers, where some animals are still purchased live, or freshly killed in markets, and freshness and quality can be assured [30,31]. This is in contrast with consumers in western cultures that more often purchase processed and packaged meat products that allow cognitive dissonance, placing a mental distance between the animal and the resulting product [32,33]. This characteristic of Chinese consumers represents an opportunity to promote animal welfare [34]. Stakeholders rated pigs as more intelligent than chickens, demonstrating that they associate intelligence with traits in humans and other mammals, such as a neo-natal appearance and in particular relatively large eyes [35].

#### Demographic Effects

In regards to the demographic effects, the residential zone in which the stakeholder lives was frequently significantly related to their attitudes, with city centre residents more likely to have clearer opinions on animal welfare, and to ‘strongly agree’ that animal welfare is important, that they have tried to make changes, and they are confident they can do so. This finding was not reflected in the previous study [15], however this may be due to a change in the nature of the questions to be more animal focussed, rather than on the driving factors of attitudes. That is, rural residents may have more regular contact with the animals, and therefore be more desensitised to animal welfare. The other explanation may be that rural residents associate with the farming environment and culture more, may have an appreciation for the complexity of farming systems and are therefore more hesitant to take a negative positions towards the welfare of animals in farming systems.

Stakeholders working within indoor intensive systems were less likely to have confidence in their ability to improve animal welfare, while the larger farms were more associated with this confidence. Systems that are highly intensive often have limited opportunity to improve welfare, in the domains of space and natural behaviour specifically, however it may be considered unexpected that size of farm had the opposite effect. Despite conventional beliefs that large farms have poor animal welfare, a scientific literature review has shown that the relationship is not simple in western farms, and that larger farms often have opportunities to improve animal welfare that smaller farms may not [36]. This may also be the case in China. Larger farms may represent larger businesses, within which stakeholders feel they have more scope and power to influence positive animal welfare change in general. Despite this stakeholders involved with very large farms were less likely to agree or strongly agree that they intended to make welfare improvements. Alternatively, this result may be reflective of confidence to improve animal welfare as there are perceived to be more animal welfare challenges to address and little economic imperative to do so.

### 4.3. Limitations of the Study

Stakeholders within this study responded to many questions with neutral responses, with strong agreement in only a few questions, and only subtle disagreement where it existed. This may indicate that responses were approached with caution and moderation, which is believed to reflect a cultural tendency to take less extreme positions and preference for the middle ground, where this may vary substantially in other cultures [15,37]. Other empirical reasons for a prevalence of neutral responses that may be relevant in this study include an avoidance of negativity associated with conflicting feelings on an issue [38], avoiding the cognitive effort required to choose between positive and negative feelings on a complicated issue [39], and a reluctance to voice a socially undesirable opinion [40]. In any case, it can be inferred that where noticeable and consistent agreement or disagreement exists, the finding is significant.

## 5. Conclusions

This study shows that stakeholders in the Chinese pig and poultry industries tended to agree that animal welfare is important to them, but that the recognition and intention to improve it was stronger in stakeholders in small and outdoor farms than in intensive indoor farms. This may be associated to the perceived costs in transitioning from a strictly intensive system to a system that allows for improved animal welfare.

## Figures and Tables

**Table 1:** Description of respondent demographics.

Demographic	Variable	N	%
**Gender**	Male	105	58.99
	Female	73	41.01
**Residential zone**	Rural	71	39.89
	Town	23	12.92
	Suburban	19	10.67
	City Centre	65	36.52
**Age**	Under 18	4	2.25
	18–25	33	18.54
	26–35	68	38.2
	36–45	58	32.58
	46–55	12	6.74
	56–65	2	1.12
	Over 65	1	0.56
**Role in industry**	Working directly with animals	44	24.72
	Team leader	5	2.81
	Business owner or manager	26	14.61
	Livestock veterinarian	10	5.62
	Government representative	13	7.3
	Other	80	44.94
**Farming system used (pig)**	Intensive indoor	116	65.2
	Indoor/outdoor	20	11.2
	Outdoor sties	14	7.9
	Outdoor free range	24	13.5
	Not involved in pig farming	4	2.2
**Size of farm (no. pigs)**	1–10	21	12.5
	10–50	8	4.76
	50–100	18	10.71
	100–500	30	17.86
	500–3000	11	6.55
	3000+	80	47.62
**Farming system used (chicken)**	Indoor enriched	45	25.28
	Indoor intensive	21	11.8
	Outdoor coup	11	6.18
	Outdoor free range	9	5.06
	Outdoor day/coup night	13	7.3
	Not involved in chicken farming	79	44.38
**Size of farm (no. chickens)**	1–50	46	31.51
	50–200	14	9.59
	200–1000	16	10.96
	1000–10,000	22	15.07
	10,000+	48	32.88
How knowledge gained *	Formal qualifications in agriculture	72	26.37
	Hands-on farm employment	88	32.23
	Personal interest (internet, journals, newspaper, television)	57	20.87
	Peers	33	12.08
	All of the above	23	8.42
**Length of time in industry**	<1 year	77	43.26
	2–3 years	25	14.04
	3–5 years	22	12.36
	5–9 years	34	19.1
	10–15 years	13	7.3
	>15 years	7	3.93

* This demographic criteria was open to multiple responses, therefore the total count is greater than the number of respondents.

**Table 2:** Responses to general attitude questions about animal welfare, with differences between mean responses.

	Likert Scale Response					Mean Response ^1^
	Strongly Disagree	Disagree	Neither Disagree nor Agree	Agree	Strongly Agree	
The welfare of animals is important to me	1	2	33	90	40	3.92
I intend to make improvements to the welfare of the animals in my care	0	2	55	98	23	3.79
In the past I have tried to make improvements to the welfare of the animals in my care	0	3	58	99	18	3.74
I am confident that I can make improvements to the welfare of animals	1	8	53	95	21	3.71
Most people who are important to me would approve of me making improvements to the welfare of the animals in my care	0	8	68	82	20	3.64
The welfare of farm animals is satisfactory in my workplace	3	18	81	63	13	3.36

^1^ Mean responses were calculated by attributing numbers 1–5 for ‘strongly disagree’ to ‘strongly agree’, respectively.

**Table 3:** Responses to attitude questions about the impact of type of pig and chicken farming system on animal welfare, with differences between mean responses.

	Likert Scale Response					Mean Response ^1^
	Strongly Disagree	Disagree	Neither Disagree nor Agree	Agree	Strongly Agree	
PIG FARMING SYSTEMS						
I believe indoor intensive farming of pigs is better for food safety	9	23	67	67	12	3.28
I believe indoor intensive farming systems are better for the welfare of the pigs	8	42	75	39	14	3.05
I believe outdoor farming systems where pigs are in groups and able to roam are better for the welfare of the pig	4	7	34	105	28	3.82
I believe housing sows in individual stalls is better for the welfare of the sow	3	22	56	75	22	3.51
I believe it is desirable to house pigs in a group situation	4	17	65	75	17	3.47
CHICKEN FARMING SYSTEMS						
I believe indoor intensive farming of meat chickens is better for food safety	10	27	57	68	16	3.29
I believe indoor intensive farming systems are better for the welfare of the chicken	11	46	66	44	11	2.98
I believe outdoor farming systems where chickens are in groups and able to roam are better for the welfare of the chicken	1	6	47	98	26	3.79

^1^ Mean responses were calculated by attributing numbers 1–5 for ‘strongly disagree’ to ‘strongly agree’, respectively.

**Table 4:** Attitudes towards pigs and their behaviour, with differences between mean responses.

	Likert Response					Mean Response ^1^
	Strongly Disagree	Disagree	Neither Disagree or Agree	Agree	Strongly Agree	
It is important that pigs are physically healthy	0	4	29	107	38	4.00
It is important that the pigs are happy	1	4	39	102	32	3.89
Pigs are intelligent animals	0	4	42	99	32	3.88
I believe pigs feel pain	1	9	44	99	25	3.77
Pigs are social animals	0	8	56	93	21	3.71
I believe pigs can suffer	1	8	62	87	20	3.65
Sows should be allowed to make a nest before they give birth	0	13	51	95	19	3.67
Pigs should be allowed to forage for food and root in the ground	3	20	59	85	11	3.45
Pigs are aggressive animals	7	33	62	68	8	3.20
It is normal for pigs to display mouthing behaviours such as biting bars and chewing (without food) repetitively	19	42	55	55	7	2.93
Pigs do not understand much about their environment	26	75	44	29	4	2.49
Piglets do not mind if their tails are docked and teeth clipped	27	78	42	27	4	2.45
Pigs are unfriendly animals and do not like to interact with other pigs	23	85	47	19	4	2.41

^1^ Mean responses were calculated by attributing numbers 1–5 for strongly disagree to strongly agree, respectively.

**Table 5:** Attitude towards chickens and their behaviour, with differences between mean responses.

	Likert Scale Response					Mean Response ^1^
	Strongly Disagree	Disagree	Neither Disagree nor Agree	Agree	Strongly Agree	
It is important that chickens are physically healthy	0	2	28	113	35	4.01
It is important that the chickens are happy	2	2	35	106	33	3.93
Chickens should be allowed to perch	1	2	37	113	25	3.89
Chickens should be allowed to stretch and preen	0	4	38	108	28	3.89
I believe chickens feel pain	0	5	36	112	25	3.88
Chickens should be allowed to flap their wings	1	6	36	114	21	3.83
Hens should be allowed to make a nest to lay their eggs in	1	8	38	112	19	3.78
Chickens are social animals	1	6	62	88	21	3.68
Chickens should be allowed to peck the dirt to forage for food	1	14	45	99	19	3.67
Chickens are intelligent animals	1	8	54	99	16	3.67
Chickens should be allowed to bathe in dust or dirt	2	18	52	87	19	3.57
I believe chickens can suffer	0	19	64	75	20	3.53
It is normal for chickens to attack each other	18	43	51	57	9	2.97
It is normal behaviour for chickens to peck each other’s feathers out	21	50	44	54	9	2.88
Chickens do not care much about their environment	28	67	54	24	5	2.5
Chickens are not friendly and do not like to interact with other chickens	23	86	46	19	4	2.41

^1^ Mean responses were calculated by attributing numbers 1–5 for strongly disagree to strongly agree, respectively.
